# 2,2,5,7,8-Penta­methyl­chroman-6-yl 2,3,4,6-tetra-*O*-acetyl-α-d-glucopyran­oside from synchrotron data

**DOI:** 10.1107/S160053681100626X

**Published:** 2011-02-26

**Authors:** Krzysztof Brzezinski, Piotr Wałejko, Aneta Baj, Stanisław Witkowski, Zbigniew Dauter

**Affiliations:** a Synchrotron Radiation Research Section, MCL, National Cancer Institute, Argonne National Laboratory, Biosciences Division, Bldg 202, Argonne, IL 60439, USA; b Institute of Chemistry, University of Białystok, Piłsudskiego 11/4, 15-443 Białystok, Poland

## Abstract

The crystal structure of the title compound, C_28_H_38_O_11_, solved and refined against synchrotron diffraction data, contains two formula units in the asymmetric unit. In both mol­ecules, the dihydro­pyran ring along with its methyl substituents is disordered and adopts two alternative half-chair conformations. The occupancy of the major conformers of the two mol­ecules refined to 0.858 (5) and 0.523 (5).

## Related literature

For background to the chemistry of α-tocopherol [systematic name 2,7,8-trimethyl-2-(4,8,12-trimethyltridecyl)-3,4-dihydro­chromen-6-ol] and its derivatives and their applications, see: Dubbs & Gupta (1998 ▶); Azzi & Stoker (2000 ▶); Traber & Atkinson (2007 ▶). For the preparation, see: Witkowski & Walejko (2002 ▶).
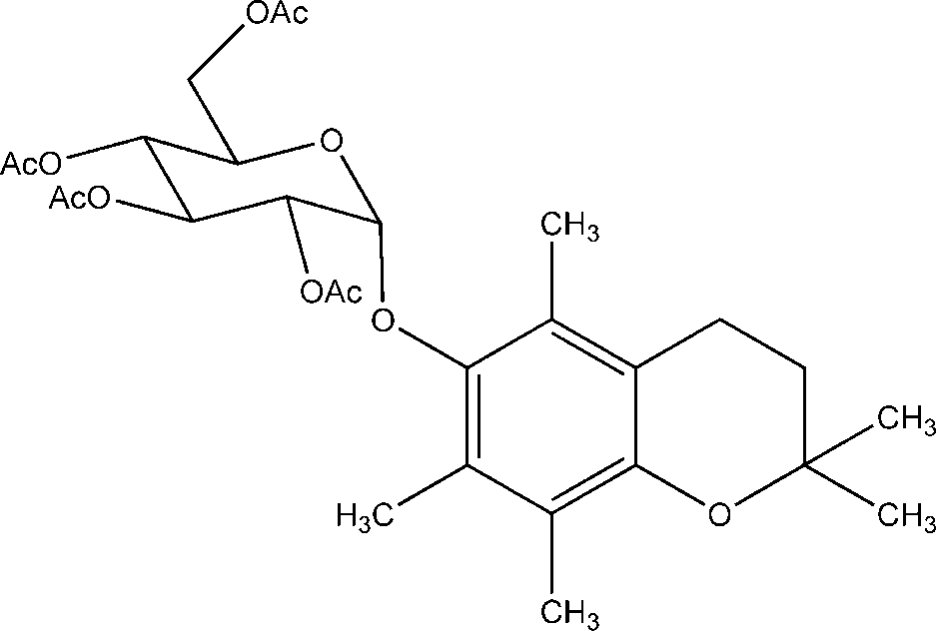


## Experimental

### 

#### Crystal data

C_28_H_38_O_11_
*M* *_r_* = 550.58
Triclinic, 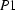

*a* = 8.66 (1) Å
*b* = 11.30 (1) Å
*c* = 14.55 (1) Å
α = 85.74 (5)°
β = 89.13 (5)°
γ = 88.16 (5)°
*V* = 1419 (2) Å^3^
*Z* = 2
Synchrotron radiation
λ = 0.59040 Å
μ = 0.06 mm^−1^
*T* = 100 K
0.25 × 0.15 × 0.09 mm

#### Data collection

Mar Research MAR315 CCD diffractometer
Absorption correction: multi-scan (*SCALEPACK*; Otwinowski & Minor, 2003 ▶) *T*_min_ = 0.985, *T*_max_ = 0.995
8538 measured reflections
5956 independent reflections
5260 reflections with *I* > 2σ(*I*)
*R*_int_ = 0.023

#### Refinement

*R*[*F* ^2^ > 2σ(*F* ^2^)] = 0.045
*wR*(*F* ^2^) = 0.118
*S* = 0.99
5956 reflections
795 parameters
752 restraints
H-atom parameters constrained
Δρ_max_ = 0.21 e Å^−3^
Δρ_min_ = −0.23 e Å^−3^

### 

Data collection: NECAT APS beamline software (unpublished); cell refinement: *HKL-2000* (Otwinowski & Minor, 1997 ▶); data reduction: *HKL-2000*; program(s) used to solve structure: *SHELXD* (Sheldrick, 2008 ▶); program(s) used to refine structure: *SHELXL97* (Sheldrick, 2008 ▶); molecular graphics: *ORTEP-3* (Farrugia, 1997 ▶) and *pyMOL* (DeLano, 2002 ▶); software used to prepare material for publication: *SHELXL97*.

## Supplementary Material

Crystal structure: contains datablocks global, I. DOI: 10.1107/S160053681100626X/gk2341sup1.cif

Structure factors: contains datablocks I. DOI: 10.1107/S160053681100626X/gk2341Isup2.hkl

Additional supplementary materials:  crystallographic information; 3D view; checkCIF report

## Figures and Tables

**Fig. 1.**
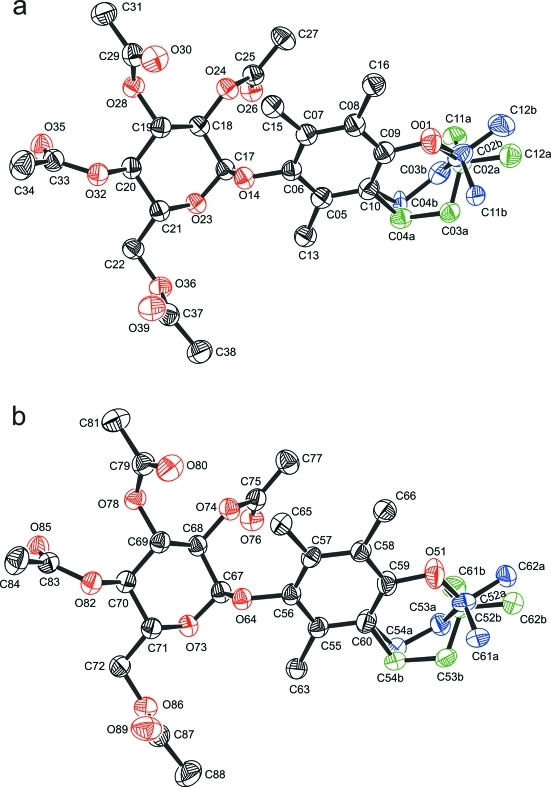
The molecular structure of the title compound. For clarity, two symmetrically independent molecules are shown separately (a and b) with hydrogen atoms omitted. Carbon atoms of the chroman systems which adopt two different conformations, are shown in green and blue, respectively. Displacement ellipsoids are drawn at the 50% probability level.

**Fig. 2.**
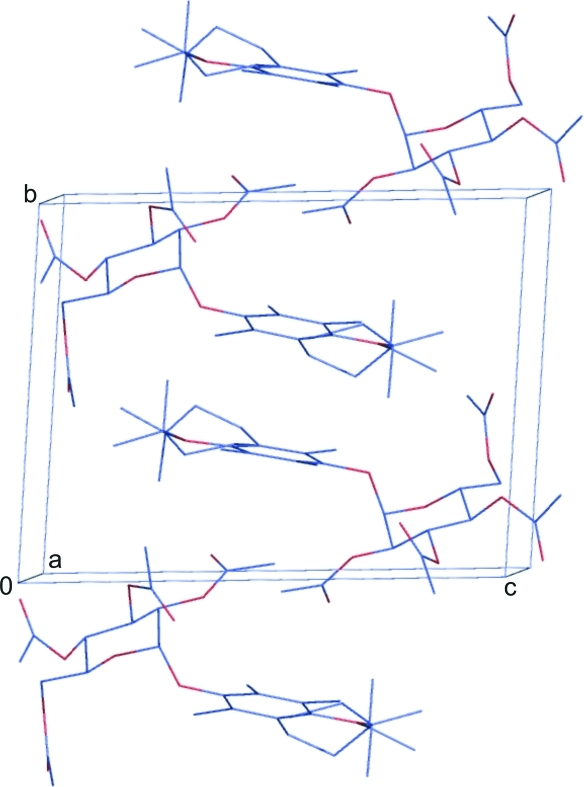
The packing diagram viewed along a axis. Only non-hydrogen atoms are shown.

## supplementary crystallographic information

### Comment

α-Tocopherol (vitamin E) is a lipophilic compound, poorly soluble in water (Dubbs & Gupta, 1998). It is also poorly absorbed after oral administration. 2,2,5,7,8-Pentamethyl-6-hydroxychroman in which the lipophylic phytyl chain was replaced with a methyl group is often used as a model compound for structural and biological investigations. Enhancing of aqueous solubility is of interest because of challenges in supplying the vitamin E preparations. In order to improve pharmacological properties, the model compound was converted into the water-soluble glucoside which is easy cleavable by the appropriate enzymes or acidic medium (Witkowski & Walejko, 2002).

In both symmetrically independent molecules, the heterocyclic ring of chroman system exists in the approximate half-chair conformation with two possible alternative positions, *exo* or *endo*, of the out of plane atom C03 (C53), as illustrated in the packing diagram, Fig. 2. As a consequence, there are also two alternative positions of the methyl substituents at C02 (C52) atoms. The occupancy ratio is 0.858:0.142 (0.005) and 0.523:0.477 (0.005), in the first and the second molecule, respectively. Two independent molecules in the cell are in the relation resembling the 2_1_ axis parallel to *a* direction, Fig. 2. This effect is emphasized by the β and γ unit-cell angles being close to 90°.

### Experimental

The title glucoside was synthesized by a modified Helferich method according to the published procedure (Witkowski & Walejko, 2002). The crystallization was carried out at room temperature by slow evaporation of 2,2,5,7,8-pentamethyl-6-hydroxychromanyl 2,3,4,6-tetra-*O*-acetyl-α-*D*-glucopyranoside solution in ethanol.

### Refinement

Fridel related reflections were averaged. The D configuration and anomeric state of the sugar moiety has been attributed according to synthesis and NMR studies (Witkowski & Walejko, 2002). Distance and angle restraints were only applied to the disordered parts of chroman moieties. All hydrogen atoms were constrained to idealized positions with C—H distances fixed at 0.98–1.00 Å and *U*_iso_(H) = 1.5*U*_eq_(C) for methyl hydrogen atoms and 1.2*U*_eq_(C) for others. The sum of occupancies of alternative positions of disordered atoms of was constrained to unity.

### Crystal data

**Table:**

C_28_H_38_O_11_	*Z* = 2
*M**_r_* = 550.58	*F*(000) = 588
Triclinic, *P*1	*D*_x_ = 1.289 Mg m^−^^3^
Hall symbol: P 1	Synchrotron radiation, λ = 0.59040 Å
*a* = 8.66 (1) Å	Cell parameters from 5956 reflections
*b* = 11.30 (1) Å	θ = 1.5–22.6°
*c* = 14.55 (1) Å	µ = 0.06 mm^−^^1^
α = 85.74 (5)°	*T* = 100 K
β = 89.13 (5)°	Needle, colourless
γ = 88.16 (5)°	0.25 × 0.15 × 0.09 mm
*V* = 1419 (2) Å^3^	

### Data collection

**Table:**

Mar Research MAR315 CCD diffractometer	5956 independent reflections
Radiation source: NECAT 24ID-C synchrotron beamline APS, USA	5260 reflections with *I* > 2σ(*I*)
Si111 double crystal	*R*_int_ = 0.023
ω scans	θ_max_ = 22.6°, θ_min_ = 1.5°
Absorption correction: multi-scan (*SCALEPACK*; Otwinowski *et al.*, 2003)	*h* = 0→11
*T*_min_ = 0.985, *T*_max_ = 0.995	*k* = −14→14
8538 measured reflections	*l* = −18→18

### Refinement

**Table:**

Refinement on *F*^2^	Primary atom site location: structure-invariant direct methods
Least-squares matrix: full	Secondary atom site location: difference Fourier map
*R*[*F*^2^ > 2σ(*F*^2^)] = 0.045	Hydrogen site location: inferred from neighbouring sites
*wR*(*F*^2^) = 0.118	H-atom parameters constrained
*S* = 0.99	*w* = 1/[σ^2^(*F*_o_^2^) + (0.0671*P*)^2^] where *P* = (*F*_o_^2^ + 2*F*_c_^2^)/3
5956 reflections	(Δ/σ)_max_ < 0.001
795 parameters	Δρ_max_ = 0.21 e Å^−^^3^
752 restraints	Δρ_min_ = −0.23 e Å^−^^3^

### Special details

**Table:**

Experimental. The crystal was mounted with vaseline on a pin attached capillary. Upon mounting, the crystal was quenched to 100 K in a nitrogen-gas stream supplied by an Oxford Cryo-Jet. Diffraction data were measured at the station 24-ID—C of the APS synchrotron by rotation method.
Geometry. All e.s.d.'s are estimated using the full covariance matrix. The cell e.s.d.'s are taken into account individually in the estimation of e.s.d.'s in distances, angles and torsion angles; correlations between e.s.d.'s in cell parameters are only used when they are defined by crystal symmetry.
Refinement. Refinement of *F*^2^ against all reflections. The weighted *R*-factor *wR* and goodness of fit *S* are based on *F*^2^, conventional *R*-factors *R* are based on *F*, with *F* set to zero for negative *F*^2^. The threshold expression of *F*^2^ > 2σ(*F*^2^) is used only for calculating *R*-factors *etc*. and is not relevant to the choice of reflections for refinement. *R*-factors based on *F*^2^ are statistically about twice as large as those based on *F*.

### Fractional atomic coordinates and isotropic or equivalent isotropic displacement parameters (Å^2^)

**Table:**

	*x*	*y*	*z*	*U*_iso_*/*U*_eq_	Occ. (<1)
O01	0.7561 (2)	0.6151 (2)	0.69759 (14)	0.0488 (5)	
C02A	0.9136 (8)	0.6057 (5)	0.7333 (3)	0.0389 (13)	0.858 (5)
C03A	1.0148 (4)	0.5345 (3)	0.6699 (2)	0.0389 (8)	0.858 (5)
HA03A	0.9747	0.4536	0.6689	0.047*	0.858 (5)
HA03B	1.1210	0.5272	0.6944	0.047*	0.858 (5)
C04A	1.0197 (5)	0.5922 (4)	0.5721 (4)	0.0409 (10)	0.858 (5)
HA04A	1.0866	0.6618	0.5697	0.049*	0.858 (5)
HA04B	1.0647	0.5350	0.5301	0.049*	0.858 (5)
C02B	0.885 (5)	0.612 (2)	0.7368 (19)	0.051 (9)	0.142 (5)
C03B	1.013 (3)	0.672 (2)	0.6797 (13)	0.064 (6)	0.142 (5)
HB03A	1.1138	0.6506	0.7090	0.077*	0.142 (5)
HB03B	0.9975	0.7594	0.6803	0.077*	0.142 (5)
C04B	1.020 (2)	0.639 (3)	0.5796 (15)	0.048 (6)	0.142 (5)
HB04A	1.0786	0.6992	0.5417	0.058*	0.142 (5)
HB04B	1.0758	0.5614	0.5765	0.058*	0.142 (5)
C05	0.8296 (3)	0.6572 (2)	0.4453 (2)	0.0394 (6)	
C06	0.6793 (3)	0.6888 (2)	0.41880 (19)	0.0353 (5)	
C07	0.5552 (3)	0.6891 (2)	0.48235 (19)	0.0366 (6)	
C08	0.5853 (3)	0.6646 (2)	0.5762 (2)	0.0392 (6)	
C09	0.7371 (3)	0.6387 (2)	0.6032 (2)	0.0400 (6)	
C10	0.8588 (3)	0.6314 (3)	0.5404 (2)	0.0415 (6)	
C11A	0.9684 (4)	0.7319 (3)	0.7378 (3)	0.0473 (9)	0.858 (5)
HA11A	0.9710	0.7715	0.6756	0.071*	0.858 (5)
HA11B	1.0722	0.7295	0.7639	0.071*	0.858 (5)
HA11C	0.8972	0.7758	0.7768	0.071*	0.858 (5)
C12A	0.8999 (4)	0.5440 (4)	0.8282 (3)	0.0474 (9)	0.858 (5)
HA12A	0.8627	0.4637	0.8235	0.071*	0.858 (5)
HA12B	0.8267	0.5888	0.8653	0.071*	0.858 (5)
HA12C	1.0013	0.5394	0.8574	0.071*	0.858 (5)
C11B	0.902 (3)	0.4772 (19)	0.7345 (14)	0.051 (6)	0.142 (5)
HB11A	0.8710	0.4392	0.7943	0.077*	0.142 (5)
HB11B	1.0103	0.4552	0.7214	0.077*	0.142 (5)
HB11C	0.8365	0.4507	0.6862	0.077*	0.142 (5)
C12B	0.892 (3)	0.646 (3)	0.8367 (16)	0.061 (7)	0.142 (5)
HB12A	0.9000	0.7324	0.8372	0.092*	0.142 (5)
HB12B	0.9819	0.6067	0.8669	0.092*	0.142 (5)
HB12C	0.7975	0.6213	0.8699	0.092*	0.142 (5)
C13	0.9577 (3)	0.6458 (3)	0.3752 (2)	0.0443 (7)	
H13A	0.9135	0.6440	0.3137	0.066*	
H13B	1.0181	0.5722	0.3901	0.066*	
H13C	1.0250	0.7138	0.3761	0.066*	
O14	0.6441 (2)	0.71514 (15)	0.32506 (13)	0.0379 (4)	
C15	0.3903 (3)	0.7082 (2)	0.4519 (2)	0.0403 (6)	
H15A	0.3880	0.7258	0.3850	0.060*	
H15B	0.3428	0.7750	0.4824	0.060*	
H15C	0.3327	0.6364	0.4685	0.060*	
C16	0.4554 (4)	0.6646 (3)	0.6465 (2)	0.0442 (6)	
H16A	0.4985	0.6602	0.7086	0.066*	
H16B	0.3913	0.5958	0.6401	0.066*	
H16C	0.3921	0.7377	0.6364	0.066*	
C17	0.7064 (3)	0.8206 (2)	0.28211 (18)	0.0366 (6)	
H17A	0.7912	0.8480	0.3205	0.044*	
C18	0.5803 (3)	0.9181 (2)	0.26962 (19)	0.0370 (6)	
H18A	0.6253	0.9914	0.2387	0.044*	
C19	0.4521 (3)	0.8772 (2)	0.21147 (19)	0.0376 (6)	
H19A	0.4010	0.8078	0.2443	0.045*	
C20	0.5187 (3)	0.8427 (2)	0.11993 (19)	0.0367 (6)	
H20A	0.5521	0.9145	0.0813	0.044*	
C21	0.6533 (3)	0.7531 (2)	0.13481 (19)	0.0369 (6)	
H21A	0.6143	0.6762	0.1632	0.044*	
C22	0.7377 (3)	0.7323 (2)	0.0457 (2)	0.0412 (6)	
H22A	0.6662	0.6991	0.0026	0.049*	
H22B	0.7735	0.8089	0.0170	0.049*	
O23	0.7644 (2)	0.79836 (16)	0.19401 (13)	0.0378 (4)	
O24	0.5137 (2)	0.94463 (16)	0.35732 (13)	0.0395 (4)	
C25	0.5971 (3)	1.0157 (2)	0.4072 (2)	0.0409 (6)	
O26	0.7141 (2)	1.05995 (18)	0.37950 (15)	0.0467 (5)	
C27	0.5227 (4)	1.0309 (3)	0.4989 (2)	0.0516 (8)	
H27A	0.4863	0.9542	0.5250	0.077*	
H27B	0.4350	1.0876	0.4915	0.077*	
H27C	0.5981	1.0609	0.5403	0.077*	
O28	0.3402 (2)	0.97388 (16)	0.19391 (13)	0.0397 (4)	
C29	0.1984 (3)	0.9609 (2)	0.2340 (2)	0.0419 (6)	
O30	0.1621 (3)	0.8758 (2)	0.28195 (16)	0.0535 (5)	
C31	0.0959 (4)	1.0682 (3)	0.2114 (3)	0.0538 (8)	
H31A	0.1392	1.1373	0.2374	0.081*	
H31B	−0.0074	1.0546	0.2378	0.081*	
H31C	0.0888	1.0828	0.1444	0.081*	
O32	0.3995 (2)	0.78273 (16)	0.07392 (13)	0.0403 (4)	
C33	0.3230 (4)	0.8441 (3)	0.0039 (2)	0.0435 (6)	
C34	0.1947 (4)	0.7725 (3)	−0.0281 (3)	0.0618 (9)	
H34A	0.1207	0.8249	−0.0630	0.093*	
H34B	0.1423	0.7331	0.0254	0.093*	
H34C	0.2371	0.7125	−0.0677	0.093*	
O35	0.3556 (3)	0.94005 (19)	−0.02650 (15)	0.0519 (5)	
O36	0.8680 (2)	0.65193 (17)	0.06139 (14)	0.0443 (5)	
C37	0.8400 (4)	0.5356 (3)	0.0729 (2)	0.0458 (7)	
C38	0.9850 (4)	0.4649 (3)	0.0940 (3)	0.0607 (9)	
H38A	1.0619	0.4824	0.0452	0.091*	
H38B	0.9632	0.3801	0.0976	0.091*	
H38C	1.0252	0.4856	0.1532	0.091*	
O39	0.7119 (3)	0.49706 (19)	0.06786 (16)	0.0533 (5)	
O51	0.2336 (2)	0.3517 (3)	0.28927 (15)	0.0623 (7)	
C52A	0.371 (2)	0.3815 (16)	0.2494 (14)	0.037 (3)	0.523 (5)
C53A	0.5086 (7)	0.3217 (6)	0.3023 (4)	0.0447 (14)	0.523 (5)
HA53A	0.5085	0.2348	0.2971	0.054*	0.523 (5)
HA53B	0.6064	0.3518	0.2750	0.054*	0.523 (5)
C54A	0.4982 (11)	0.3477 (8)	0.4039 (6)	0.0405 (19)	0.523 (5)
HA54A	0.5682	0.2912	0.4395	0.049*	0.523 (5)
HA54B	0.5352	0.4287	0.4102	0.049*	0.523 (5)
C52B	0.398 (2)	0.377 (2)	0.2549 (13)	0.038 (3)	0.477 (5)
C53B	0.4895 (7)	0.4495 (5)	0.3180 (4)	0.0389 (14)	0.477 (5)
HB53A	0.5959	0.4585	0.2932	0.047*	0.477 (5)
HB53B	0.4405	0.5297	0.3197	0.047*	0.477 (5)
C54B	0.4957 (11)	0.3904 (7)	0.4148 (7)	0.0355 (19)	0.477 (5)
HB54A	0.5254	0.4491	0.4581	0.043*	0.477 (5)
HB54B	0.5755	0.3255	0.4173	0.043*	0.477 (5)
C55	0.3091 (3)	0.3160 (2)	0.53997 (19)	0.0365 (6)	
C56	0.1604 (3)	0.2858 (2)	0.56897 (18)	0.0345 (5)	
C57	0.0357 (3)	0.2893 (2)	0.50902 (19)	0.0342 (5)	
C58	0.0645 (3)	0.3129 (2)	0.41409 (19)	0.0366 (6)	
C59	0.2153 (3)	0.3346 (3)	0.3839 (2)	0.0437 (6)	
C60	0.3370 (3)	0.3390 (3)	0.4452 (2)	0.0441 (7)	
C61A	0.3660 (7)	0.5140 (5)	0.2516 (4)	0.0457 (14)	0.523 (5)
HA61A	0.3073	0.5496	0.1990	0.069*	0.523 (5)
HA61B	0.4715	0.5429	0.2484	0.069*	0.523 (5)
HA61C	0.3159	0.5360	0.3091	0.069*	0.523 (5)
C62A	0.3814 (7)	0.3441 (6)	0.1501 (4)	0.0449 (14)	0.523 (5)
HA62A	0.3859	0.2573	0.1510	0.067*	0.523 (5)
HA62B	0.4749	0.3759	0.1201	0.067*	0.523 (5)
HA62C	0.2904	0.3752	0.1160	0.067*	0.523 (5)
C61B	0.4591 (8)	0.2526 (6)	0.2504 (5)	0.0485 (16)	0.477 (5)
HB61A	0.5649	0.2534	0.2256	0.073*	0.477 (5)
HB61B	0.3936	0.2104	0.2103	0.073*	0.477 (5)
HB61C	0.4589	0.2123	0.3124	0.073*	0.477 (5)
C62B	0.3773 (8)	0.4428 (7)	0.1606 (4)	0.0498 (17)	0.477 (5)
HB62A	0.3323	0.5223	0.1681	0.075*	0.477 (5)
HB62B	0.3082	0.3989	0.1237	0.075*	0.477 (5)
HB62C	0.4779	0.4498	0.1292	0.075*	0.477 (5)
C63	0.4369 (3)	0.3278 (2)	0.60830 (19)	0.0404 (6)	
H63A	0.4980	0.3969	0.5887	0.061*	
H63B	0.5037	0.2560	0.6109	0.061*	
H63C	0.3916	0.3380	0.6694	0.061*	
O64	0.1302 (2)	0.25883 (15)	0.66386 (12)	0.0352 (4)	
C65	−0.1279 (3)	0.2750 (2)	0.5431 (2)	0.0383 (6)	
H65A	−0.1896	0.3467	0.5242	0.058*	
H65B	−0.1291	0.2627	0.6104	0.058*	
H65C	−0.1717	0.2063	0.5167	0.058*	
C66	−0.0684 (3)	0.3201 (3)	0.3479 (2)	0.0418 (6)	
H66A	−0.1293	0.2486	0.3580	0.063*	
H66B	−0.0282	0.3261	0.2845	0.063*	
H66C	−0.1340	0.3902	0.3583	0.063*	
C67	0.1988 (3)	0.1507 (2)	0.70335 (19)	0.0370 (6)	
H67A	0.2818	0.1218	0.6613	0.044*	
C68	0.0754 (3)	0.0573 (2)	0.71880 (18)	0.0358 (5)	
H68A	0.1241	−0.0177	0.7474	0.043*	
C69	−0.0498 (3)	0.1007 (2)	0.78254 (19)	0.0368 (6)	
H69A	−0.1018	0.1741	0.7533	0.044*	
C70	0.0221 (3)	0.1284 (2)	0.87262 (19)	0.0360 (6)	
H70A	0.0622	0.0535	0.9061	0.043*	
C71	0.1532 (3)	0.2149 (2)	0.85451 (18)	0.0359 (6)	
H71A	0.1100	0.2939	0.8300	0.043*	
C72	0.2424 (3)	0.2292 (2)	0.9411 (2)	0.0404 (6)	
H72A	0.1761	0.2712	0.9851	0.049*	
H72B	0.2713	0.1499	0.9705	0.049*	
O73	0.2619 (2)	0.16994 (16)	0.78913 (12)	0.0373 (4)	
O74	0.0062 (2)	0.03338 (16)	0.63368 (13)	0.0390 (4)	
C75	0.0909 (3)	−0.0373 (2)	0.5785 (2)	0.0402 (6)	
O76	0.2155 (2)	−0.07916 (18)	0.60091 (15)	0.0470 (5)	
C77	0.0086 (4)	−0.0553 (3)	0.4925 (2)	0.0510 (7)	
H77A	0.0792	−0.0936	0.4497	0.077*	
H77B	−0.0280	0.0217	0.4642	0.077*	
H77C	−0.0797	−0.1058	0.5066	0.077*	
O78	−0.1617 (2)	0.01101 (16)	0.80209 (13)	0.0386 (4)	
C79	−0.3014 (3)	0.0277 (3)	0.7598 (2)	0.0439 (6)	
O80	−0.3347 (2)	0.1129 (2)	0.71021 (16)	0.0539 (6)	
C81	−0.4040 (4)	−0.0740 (3)	0.7847 (3)	0.0565 (8)	
H81A	−0.5076	−0.0436	0.8001	0.085*	
H81B	−0.3616	−0.1218	0.8380	0.085*	
H81C	−0.4098	−0.1234	0.7323	0.085*	
O82	−0.0937 (2)	0.18503 (15)	0.92817 (13)	0.0384 (4)	
C83	−0.1639 (3)	0.1161 (2)	0.9962 (2)	0.0396 (6)	
C84	−0.2941 (4)	0.1831 (3)	1.0383 (2)	0.0542 (8)	
H84A	−0.2727	0.2679	1.0334	0.081*	
H84B	−0.3057	0.1552	1.1034	0.081*	
H84C	−0.3898	0.1701	1.0058	0.081*	
O85	−0.1246 (3)	0.01617 (18)	1.01807 (15)	0.0516 (5)	
O86	0.3803 (2)	0.2954 (2)	0.92082 (15)	0.0484 (5)	
C87	0.3644 (4)	0.4134 (3)	0.9065 (2)	0.0524 (8)	
C88	0.5135 (5)	0.4673 (5)	0.8774 (3)	0.0841 (14)	
H88A	0.4979	0.5534	0.8660	0.126*	
H88B	0.5514	0.4336	0.8209	0.126*	
H88C	0.5895	0.4504	0.9263	0.126*	
O89	0.2423 (3)	0.4656 (2)	0.91500 (18)	0.0616 (6)	

### Atomic displacement parameters (Å^2^)

**Table:**

	*U*^11^	*U*^22^	*U*^33^	*U*^12^	*U*^13^	*U*^23^
O01	0.0416 (11)	0.0643 (14)	0.0400 (12)	−0.0051 (10)	−0.0039 (9)	0.0009 (10)
C02A	0.038 (2)	0.040 (3)	0.039 (2)	0.0009 (18)	−0.0043 (16)	−0.0008 (19)
C03A	0.0435 (17)	0.0315 (15)	0.0412 (18)	0.0003 (13)	−0.0014 (13)	−0.0001 (13)
C04A	0.042 (2)	0.033 (2)	0.047 (2)	−0.0019 (15)	0.0032 (15)	−0.0004 (17)
C02B	0.057 (17)	0.030 (13)	0.066 (16)	0.002 (12)	−0.010 (13)	−0.005 (12)
C03B	0.085 (13)	0.051 (11)	0.053 (12)	0.023 (11)	0.001 (11)	0.009 (10)
C04B	0.041 (11)	0.077 (16)	0.024 (10)	0.000 (12)	−0.009 (8)	0.011 (11)
C05	0.0466 (15)	0.0285 (12)	0.0431 (16)	−0.0058 (11)	0.0021 (12)	−0.0003 (11)
C06	0.0421 (14)	0.0265 (12)	0.0371 (14)	−0.0025 (10)	−0.0029 (11)	−0.0007 (10)
C07	0.0447 (14)	0.0275 (12)	0.0380 (14)	−0.0052 (10)	−0.0005 (11)	−0.0021 (10)
C08	0.0497 (16)	0.0302 (13)	0.0375 (15)	−0.0053 (11)	−0.0005 (12)	−0.0001 (11)
C09	0.0454 (15)	0.0340 (13)	0.0403 (16)	−0.0042 (11)	0.0002 (12)	0.0005 (11)
C10	0.0426 (14)	0.0377 (14)	0.0440 (16)	−0.0010 (11)	−0.0049 (12)	−0.0010 (12)
C11A	0.055 (2)	0.0369 (17)	0.052 (2)	−0.0079 (15)	−0.0018 (16)	−0.0083 (15)
C12A	0.0456 (19)	0.051 (2)	0.045 (2)	0.0000 (16)	−0.0002 (15)	0.0025 (16)
C11B	0.065 (14)	0.054 (13)	0.031 (10)	0.025 (10)	0.007 (9)	0.006 (9)
C12B	0.042 (11)	0.086 (18)	0.056 (14)	0.011 (12)	0.005 (10)	−0.015 (13)
C13	0.0491 (16)	0.0374 (15)	0.0459 (17)	−0.0032 (12)	0.0016 (13)	−0.0006 (12)
O14	0.0472 (10)	0.0316 (9)	0.0348 (10)	−0.0071 (8)	−0.0001 (8)	0.0004 (8)
C15	0.0444 (15)	0.0362 (14)	0.0404 (15)	−0.0043 (11)	0.0011 (11)	−0.0023 (11)
C16	0.0495 (16)	0.0403 (15)	0.0428 (16)	−0.0012 (12)	0.0008 (12)	−0.0034 (12)
C17	0.0456 (15)	0.0307 (13)	0.0330 (14)	−0.0055 (11)	0.0003 (11)	0.0019 (10)
C18	0.0435 (14)	0.0319 (13)	0.0359 (14)	−0.0055 (11)	0.0020 (11)	−0.0025 (11)
C19	0.0430 (14)	0.0287 (12)	0.0410 (15)	−0.0021 (11)	−0.0035 (11)	−0.0008 (11)
C20	0.0449 (15)	0.0286 (13)	0.0371 (14)	−0.0070 (11)	−0.0059 (11)	−0.0019 (10)
C21	0.0412 (14)	0.0336 (13)	0.0360 (14)	−0.0042 (11)	−0.0011 (11)	−0.0009 (11)
C22	0.0487 (16)	0.0344 (14)	0.0402 (15)	−0.0017 (12)	0.0022 (12)	−0.0022 (11)
O23	0.0419 (10)	0.0358 (10)	0.0361 (10)	−0.0060 (8)	0.0008 (8)	−0.0039 (8)
O24	0.0487 (11)	0.0308 (9)	0.0398 (11)	−0.0077 (8)	0.0009 (8)	−0.0065 (8)
C25	0.0525 (16)	0.0282 (13)	0.0427 (16)	−0.0037 (12)	−0.0019 (12)	−0.0056 (11)
O26	0.0496 (12)	0.0398 (11)	0.0516 (13)	−0.0096 (9)	−0.0027 (9)	−0.0066 (9)
C27	0.068 (2)	0.0439 (17)	0.0453 (17)	−0.0103 (15)	0.0048 (15)	−0.0139 (13)
O28	0.0440 (10)	0.0308 (9)	0.0439 (11)	−0.0009 (8)	−0.0013 (8)	0.0001 (8)
C29	0.0472 (15)	0.0375 (15)	0.0421 (16)	−0.0078 (12)	−0.0043 (12)	−0.0062 (12)
O30	0.0516 (12)	0.0491 (12)	0.0587 (14)	−0.0070 (10)	0.0070 (10)	0.0045 (10)
C31	0.0489 (17)	0.0507 (18)	0.062 (2)	0.0046 (14)	−0.0029 (14)	−0.0087 (15)
O32	0.0483 (11)	0.0307 (9)	0.0423 (11)	−0.0031 (8)	−0.0080 (8)	−0.0026 (8)
C33	0.0524 (16)	0.0374 (15)	0.0408 (15)	0.0018 (12)	−0.0064 (12)	−0.0027 (12)
C34	0.060 (2)	0.0548 (19)	0.071 (2)	−0.0059 (16)	−0.0217 (17)	−0.0072 (17)
O35	0.0635 (14)	0.0443 (12)	0.0470 (13)	−0.0027 (10)	−0.0102 (10)	0.0049 (10)
O36	0.0431 (10)	0.0422 (11)	0.0478 (12)	−0.0019 (9)	0.0010 (8)	−0.0047 (9)
C37	0.0570 (18)	0.0419 (16)	0.0381 (16)	−0.0010 (14)	0.0017 (13)	−0.0007 (12)
C38	0.065 (2)	0.060 (2)	0.055 (2)	0.0152 (17)	0.0055 (16)	0.0012 (16)
O39	0.0559 (13)	0.0428 (12)	0.0616 (15)	−0.0070 (10)	0.0014 (10)	−0.0045 (10)
O51	0.0351 (11)	0.112 (2)	0.0362 (12)	−0.0017 (12)	0.0016 (9)	0.0153 (12)
C52A	0.031 (7)	0.029 (4)	0.051 (5)	0.000 (4)	−0.005 (4)	−0.003 (3)
C53A	0.041 (3)	0.057 (4)	0.036 (3)	−0.005 (3)	−0.003 (2)	0.000 (3)
C54A	0.040 (4)	0.042 (5)	0.039 (4)	−0.007 (4)	0.002 (3)	−0.002 (3)
C52B	0.027 (6)	0.050 (6)	0.037 (6)	−0.009 (4)	0.010 (4)	0.001 (4)
C53B	0.038 (3)	0.034 (3)	0.044 (3)	−0.010 (2)	0.000 (2)	0.000 (2)
C54B	0.032 (3)	0.032 (4)	0.042 (4)	−0.004 (3)	−0.001 (3)	−0.003 (3)
C55	0.0381 (13)	0.0325 (13)	0.0382 (15)	−0.0011 (10)	−0.0034 (11)	0.0033 (11)
C56	0.0429 (14)	0.0275 (12)	0.0325 (14)	−0.0001 (10)	0.0016 (11)	0.0018 (10)
C57	0.0375 (13)	0.0279 (12)	0.0366 (14)	−0.0020 (10)	0.0017 (10)	0.0023 (10)
C58	0.0370 (14)	0.0347 (13)	0.0376 (15)	−0.0029 (11)	0.0001 (10)	0.0020 (11)
C59	0.0416 (14)	0.0523 (17)	0.0353 (15)	0.0005 (13)	−0.0034 (11)	0.0088 (12)
C60	0.0378 (14)	0.0516 (17)	0.0410 (16)	−0.0032 (12)	0.0001 (11)	0.0100 (13)
C61A	0.053 (3)	0.039 (3)	0.046 (3)	−0.017 (2)	−0.003 (3)	−0.003 (2)
C62A	0.041 (3)	0.061 (4)	0.033 (3)	−0.009 (3)	0.001 (2)	−0.004 (3)
C61B	0.053 (4)	0.044 (3)	0.050 (4)	−0.003 (3)	0.003 (3)	−0.012 (3)
C62B	0.055 (4)	0.057 (4)	0.038 (3)	−0.018 (3)	0.001 (3)	−0.001 (3)
C63	0.0436 (15)	0.0379 (14)	0.0394 (15)	−0.0039 (12)	−0.0051 (11)	0.0004 (11)
O64	0.0412 (10)	0.0301 (9)	0.0337 (10)	0.0015 (7)	0.0003 (7)	0.0004 (7)
C65	0.0382 (13)	0.0368 (14)	0.0396 (15)	−0.0026 (11)	0.0019 (11)	0.0002 (11)
C66	0.0377 (14)	0.0443 (15)	0.0429 (16)	−0.0021 (12)	−0.0041 (11)	0.0015 (12)
C67	0.0443 (15)	0.0299 (13)	0.0362 (14)	0.0023 (11)	0.0001 (11)	−0.0001 (11)
C68	0.0440 (14)	0.0302 (12)	0.0328 (14)	−0.0004 (10)	0.0004 (11)	−0.0005 (10)
C69	0.0436 (14)	0.0277 (12)	0.0386 (15)	−0.0018 (10)	0.0046 (11)	−0.0009 (10)
C70	0.0408 (14)	0.0288 (12)	0.0378 (14)	0.0025 (10)	0.0061 (11)	−0.0025 (10)
C71	0.0400 (14)	0.0298 (13)	0.0377 (15)	0.0006 (11)	0.0028 (11)	−0.0030 (10)
C72	0.0474 (15)	0.0350 (14)	0.0386 (15)	−0.0035 (12)	−0.0005 (12)	0.0001 (11)
O73	0.0394 (10)	0.0377 (10)	0.0345 (10)	0.0022 (8)	0.0005 (8)	−0.0023 (8)
O74	0.0472 (10)	0.0335 (9)	0.0364 (10)	0.0013 (8)	0.0014 (8)	−0.0056 (8)
C75	0.0511 (17)	0.0276 (13)	0.0419 (16)	−0.0015 (12)	0.0079 (12)	−0.0037 (11)
O76	0.0495 (12)	0.0390 (11)	0.0528 (13)	0.0044 (9)	0.0010 (9)	−0.0087 (9)
C77	0.064 (2)	0.0446 (16)	0.0454 (18)	0.0022 (14)	−0.0022 (14)	−0.0091 (14)
O78	0.0425 (10)	0.0315 (9)	0.0415 (11)	−0.0038 (8)	−0.0005 (8)	0.0009 (8)
C79	0.0415 (15)	0.0460 (16)	0.0438 (16)	−0.0025 (12)	−0.0012 (12)	0.0000 (13)
O80	0.0455 (12)	0.0523 (13)	0.0617 (14)	0.0003 (10)	−0.0047 (10)	0.0104 (11)
C81	0.0499 (17)	0.0492 (18)	0.070 (2)	−0.0116 (15)	−0.0026 (15)	0.0007 (16)
O82	0.0431 (10)	0.0305 (9)	0.0413 (11)	0.0005 (8)	0.0068 (8)	−0.0023 (8)
C83	0.0434 (14)	0.0361 (14)	0.0395 (15)	−0.0072 (12)	0.0023 (11)	−0.0032 (11)
C84	0.0579 (19)	0.0458 (17)	0.058 (2)	−0.0022 (14)	0.0196 (15)	−0.0041 (14)
O85	0.0649 (14)	0.0396 (11)	0.0483 (13)	0.0003 (10)	0.0079 (10)	0.0064 (9)
O86	0.0447 (11)	0.0559 (13)	0.0455 (12)	−0.0094 (10)	−0.0035 (9)	−0.0048 (10)
C87	0.058 (2)	0.057 (2)	0.0420 (17)	−0.0141 (16)	−0.0108 (14)	0.0056 (14)
C88	0.075 (3)	0.113 (4)	0.063 (3)	−0.049 (3)	−0.016 (2)	0.019 (2)
O89	0.0707 (17)	0.0452 (13)	0.0693 (17)	−0.0071 (12)	−0.0181 (12)	−0.0014 (11)

### Geometric parameters (Å, °)

**Table:**

O01—C02B	1.26 (4)	O51—C52A	1.36 (2)
O01—C09	1.390 (4)	O51—C59	1.383 (4)
O01—C02A	1.465 (7)	O51—C52B	1.53 (2)
C02A—C12A	1.504 (6)	C52A—C61A	1.499 (19)
C02A—C03A	1.518 (6)	C52A—C62A	1.53 (2)
C02A—C11A	1.524 (7)	C52A—C53A	1.541 (15)
C03A—C04A	1.521 (6)	C53A—C54A	1.529 (9)
C03A—HA03A	0.9900	C53A—HA53A	0.9900
C03A—HA03B	0.9900	C53A—HA53B	0.9900
C04A—C10	1.517 (5)	C54A—C60	1.515 (9)
C04A—HA04A	0.9900	C54A—HA54A	0.9900
C04A—HA04B	0.9900	C54A—HA54B	0.9900
C02B—C11B	1.526 (19)	C52B—C61B	1.49 (2)
C02B—C03B	1.526 (19)	C52B—C53B	1.521 (16)
C02B—C12B	1.535 (19)	C52B—C62B	1.52 (2)
C03B—C04B	1.532 (17)	C53B—C54B	1.514 (11)
C03B—HB03A	0.9900	C53B—HB53A	0.9900
C03B—HB03B	0.9900	C53B—HB53B	0.9900
C04B—C10	1.525 (17)	C54B—C60	1.554 (10)
C04B—HB04A	0.9900	C54B—HB54A	0.9900
C04B—HB04B	0.9900	C54B—HB54B	0.9900
C05—C06	1.391 (4)	C55—C56	1.394 (4)
C05—C10	1.417 (4)	C55—C60	1.403 (4)
C05—C13	1.504 (4)	C55—C63	1.514 (4)
C06—O14	1.410 (3)	C56—C57	1.396 (4)
C06—C07	1.407 (4)	C56—O64	1.414 (3)
C07—C08	1.400 (4)	C57—C58	1.407 (4)
C07—C15	1.506 (4)	C57—C65	1.504 (4)
C08—C09	1.394 (4)	C58—C59	1.395 (4)
C08—C16	1.508 (4)	C58—C66	1.508 (4)
C09—C10	1.389 (4)	C59—C60	1.396 (4)
C11A—HA11A	0.9800	C61A—HA61A	0.9800
C11A—HA11B	0.9800	C61A—HA61B	0.9800
C11A—HA11C	0.9800	C61A—HA61C	0.9800
C12A—HA12A	0.9800	C62A—HA62A	0.9800
C12A—HA12B	0.9800	C62A—HA62B	0.9800
C12A—HA12C	0.9800	C62A—HA62C	0.9800
C11B—HB11A	0.9800	C61B—HB61A	0.9800
C11B—HB11B	0.9800	C61B—HB61B	0.9800
C11B—HB11C	0.9800	C61B—HB61C	0.9800
C12B—HB12A	0.9800	C62B—HB62A	0.9800
C12B—HB12B	0.9800	C62B—HB62B	0.9800
C12B—HB12C	0.9800	C62B—HB62C	0.9800
C13—H13A	0.9800	C63—H63A	0.9800
C13—H13B	0.9800	C63—H63B	0.9800
C13—H13C	0.9800	C63—H63C	0.9800
O14—C17	1.422 (3)	O64—C67	1.426 (3)
C15—H15A	0.9800	C65—H65A	0.9800
C15—H15B	0.9800	C65—H65B	0.9800
C15—H15C	0.9800	C65—H65C	0.9800
C16—H16A	0.9800	C66—H66A	0.9800
C16—H16B	0.9800	C66—H66B	0.9800
C16—H16C	0.9800	C66—H66C	0.9800
C17—O23	1.407 (3)	C67—O73	1.404 (3)
C17—C18	1.530 (4)	C67—C68	1.529 (4)
C17—H17A	1.0000	C67—H67A	1.0000
C18—O24	1.440 (3)	C68—O74	1.432 (3)
C18—C19	1.510 (4)	C68—C69	1.510 (3)
C18—H18A	1.0000	C68—H68A	1.0000
C19—O28	1.447 (3)	C69—O78	1.435 (3)
C19—C20	1.516 (4)	C69—C70	1.517 (4)
C19—H19A	1.0000	C69—H69A	1.0000
C20—O32	1.451 (3)	C70—O82	1.442 (3)
C20—C21	1.526 (4)	C70—C71	1.530 (4)
C20—H20A	1.0000	C70—H70A	1.0000
C21—O23	1.433 (3)	C71—O73	1.436 (3)
C21—C22	1.508 (4)	C71—C72	1.509 (4)
C21—H21A	1.0000	C71—H71A	1.0000
C22—O36	1.435 (4)	C72—O86	1.445 (4)
C22—H22A	0.9900	C72—H72A	0.9900
C22—H22B	0.9900	C72—H72B	0.9900
O24—C25	1.354 (3)	O74—C75	1.364 (3)
C25—O26	1.196 (3)	C75—O76	1.204 (4)
C25—C27	1.491 (4)	C75—C77	1.480 (4)
C27—H27A	0.9800	C77—H77A	0.9800
C27—H27B	0.9800	C77—H77B	0.9800
C27—H27C	0.9800	C77—H77C	0.9800
O28—C29	1.359 (4)	O78—C79	1.367 (3)
C29—O30	1.193 (3)	C79—O80	1.190 (4)
C29—C31	1.499 (4)	C79—C81	1.495 (4)
C31—H31A	0.9800	C81—H81A	0.9800
C31—H31B	0.9800	C81—H81B	0.9800
C31—H31C	0.9800	C81—H81C	0.9800
O32—C33	1.358 (4)	O82—C83	1.361 (3)
C33—O35	1.181 (4)	C83—O85	1.190 (4)
C33—C34	1.496 (5)	C83—C84	1.488 (4)
C34—H34A	0.9800	C84—H84A	0.9800
C34—H34B	0.9800	C84—H84B	0.9800
C34—H34C	0.9800	C84—H84C	0.9800
O36—C37	1.342 (4)	O86—C87	1.337 (4)
C37—O39	1.211 (4)	C87—O89	1.204 (4)
C37—C38	1.490 (5)	C87—C88	1.488 (5)
C38—H38A	0.9800	C88—H88A	0.9800
C38—H38B	0.9800	C88—H88B	0.9800
C38—H38C	0.9800	C88—H88C	0.9800
			
C02B—O01—C09	123.9 (11)	C52A—O51—C59	122.0 (7)
C02B—O01—C02A	6.8 (12)	C52A—O51—C52B	7.3 (13)
C09—O01—C02A	118.2 (3)	C59—O51—C52B	115.5 (6)
O01—C02A—C12A	105.5 (4)	O51—C52A—C61A	102.3 (13)
O01—C02A—C03A	109.1 (4)	O51—C52A—C62A	111.0 (10)
C12A—C02A—C03A	111.8 (4)	C61A—C52A—C62A	111.4 (14)
O01—C02A—C11A	106.9 (4)	O51—C52A—C53A	112.0 (14)
C12A—C02A—C11A	111.0 (4)	C61A—C52A—C53A	112.7 (10)
C03A—C02A—C11A	112.1 (4)	C62A—C52A—C53A	107.5 (13)
C02A—C03A—C04A	112.0 (3)	C54A—C53A—C52A	110.2 (9)
C02A—C03A—HA03A	109.2	C54A—C53A—HA53A	109.6
C04A—C03A—HA03A	109.2	C52A—C53A—HA53A	109.6
C02A—C03A—HA03B	109.2	C54A—C53A—HA53B	109.6
C04A—C03A—HA03B	109.2	C52A—C53A—HA53B	109.6
HA03A—C03A—HA03B	107.9	HA53A—C53A—HA53B	108.1
C10—C04A—C03A	110.8 (3)	C60—C54A—C53A	114.0 (6)
C10—C04A—HA04A	109.5	C60—C54A—HA54A	108.8
C03A—C04A—HA04A	109.5	C53A—C54A—HA54A	108.8
C10—C04A—HA04B	109.5	C60—C54A—HA54B	108.8
C03A—C04A—HA04B	109.5	C53A—C54A—HA54B	108.8
HA04A—C04A—HA04B	108.1	HA54A—C54A—HA54B	107.6
O01—C02B—C11B	92.6 (19)	C61B—C52B—O51	99.3 (13)
O01—C02B—C03B	114 (2)	C61B—C52B—C53B	113.7 (12)
C11B—C02B—C03B	110 (2)	O51—C52B—C53B	114.0 (14)
O01—C02B—C12B	119 (2)	C61B—C52B—C62B	113.4 (13)
C11B—C02B—C12B	110 (2)	O51—C52B—C62B	104.6 (11)
C03B—C02B—C12B	110 (2)	C53B—C52B—C62B	111.0 (14)
C02B—C03B—C04B	113.4 (18)	C54B—C53B—C52B	111.1 (9)
C02B—C03B—HB03A	108.9	C54B—C53B—HB53A	109.4
C04B—C03B—HB03A	108.9	C52B—C53B—HB53A	109.4
C02B—C03B—HB03B	108.9	C54B—C53B—HB53B	109.4
C04B—C03B—HB03B	108.9	C52B—C53B—HB53B	109.4
HB03A—C03B—HB03B	107.7	HB53A—C53B—HB53B	108.0
C10—C04B—C03B	111.4 (16)	C53B—C54B—C60	111.2 (7)
C10—C04B—HB04A	109.3	C53B—C54B—HB54A	109.4
C03B—C04B—HB04A	109.3	C60—C54B—HB54A	109.4
C10—C04B—HB04B	109.3	C53B—C54B—HB54B	109.4
C03B—C04B—HB04B	109.3	C60—C54B—HB54B	109.4
HB04A—C04B—HB04B	108.0	HB54A—C54B—HB54B	108.0
C06—C05—C10	118.3 (3)	C56—C55—C60	118.2 (2)
C06—C05—C13	121.3 (3)	C56—C55—C63	121.5 (2)
C10—C05—C13	120.3 (3)	C60—C55—C63	120.3 (2)
C05—C06—O14	120.6 (2)	C55—C56—C57	122.8 (2)
C05—C06—C07	122.2 (3)	C55—C56—O64	119.3 (2)
O14—C06—C07	117.1 (2)	C57—C56—O64	117.7 (2)
C08—C07—C06	119.0 (3)	C56—C57—C58	118.4 (2)
C08—C07—C15	119.0 (2)	C56—C57—C65	122.2 (2)
C06—C07—C15	121.9 (2)	C58—C57—C65	119.3 (2)
C09—C08—C07	118.7 (3)	C59—C58—C57	118.9 (2)
C09—C08—C16	120.8 (3)	C59—C58—C66	121.4 (3)
C07—C08—C16	120.5 (3)	C57—C58—C66	119.6 (2)
O01—C09—C10	122.6 (3)	O51—C59—C58	114.8 (2)
O01—C09—C08	114.7 (2)	O51—C59—C60	123.2 (3)
C10—C09—C08	122.6 (3)	C58—C59—C60	122.0 (3)
C09—C10—C05	119.0 (3)	C59—C60—C55	119.3 (3)
C09—C10—C04A	120.8 (3)	C59—C60—C54A	117.0 (4)
C05—C10—C04A	120.1 (3)	C55—C60—C54A	122.8 (4)
C09—C10—C04B	115.9 (9)	C59—C60—C54B	122.1 (4)
C05—C10—C04B	121.7 (10)	C55—C60—C54B	117.6 (4)
C04A—C10—C04B	20.7 (12)	C54A—C60—C54B	19.4 (3)
C02A—C11A—HA11A	109.5	C52A—C61A—HA61A	109.5
C02A—C11A—HA11B	109.5	C52A—C61A—HA61B	109.5
HA11A—C11A—HA11B	109.5	HA61A—C61A—HA61B	109.5
C02A—C11A—HA11C	109.5	C52A—C61A—HA61C	109.5
HA11A—C11A—HA11C	109.5	HA61A—C61A—HA61C	109.5
HA11B—C11A—HA11C	109.5	HA61B—C61A—HA61C	109.5
C02A—C12A—HA12A	109.5	C52A—C62A—HA62A	109.5
C02A—C12A—HA12B	109.5	C52A—C62A—HA62B	109.5
HA12A—C12A—HA12B	109.5	HA62A—C62A—HA62B	109.5
C02A—C12A—HA12C	109.5	C52A—C62A—HA62C	109.5
HA12A—C12A—HA12C	109.5	HA62A—C62A—HA62C	109.5
HA12B—C12A—HA12C	109.5	HA62B—C62A—HA62C	109.5
C02B—C11B—HB11A	109.5	C52B—C61B—HB61A	109.5
C02B—C11B—HB11B	109.5	C52B—C61B—HB61B	109.5
HB11A—C11B—HB11B	109.5	HB61A—C61B—HB61B	109.5
C02B—C11B—HB11C	109.5	C52B—C61B—HB61C	109.5
HB11A—C11B—HB11C	109.5	HB61A—C61B—HB61C	109.5
HB11B—C11B—HB11C	109.5	HB61B—C61B—HB61C	109.5
C02B—C12B—HB12A	109.5	C52B—C62B—HB62A	109.5
C02B—C12B—HB12B	109.5	C52B—C62B—HB62B	109.5
HB12A—C12B—HB12B	109.5	HB62A—C62B—HB62B	109.5
C02B—C12B—HB12C	109.5	C52B—C62B—HB62C	109.5
HB12A—C12B—HB12C	109.5	HB62A—C62B—HB62C	109.5
HB12B—C12B—HB12C	109.5	HB62B—C62B—HB62C	109.5
C05—C13—H13A	109.5	C55—C63—H63A	109.5
C05—C13—H13B	109.5	C55—C63—H63B	109.5
H13A—C13—H13B	109.5	H63A—C63—H63B	109.5
C05—C13—H13C	109.5	C55—C63—H63C	109.5
H13A—C13—H13C	109.5	H63A—C63—H63C	109.5
H13B—C13—H13C	109.5	H63B—C63—H63C	109.5
C06—O14—C17	116.4 (2)	C56—O64—C67	116.09 (18)
C07—C15—H15A	109.5	C57—C65—H65A	109.5
C07—C15—H15B	109.5	C57—C65—H65B	109.5
H15A—C15—H15B	109.5	H65A—C65—H65B	109.5
C07—C15—H15C	109.5	C57—C65—H65C	109.5
H15A—C15—H15C	109.5	H65A—C65—H65C	109.5
H15B—C15—H15C	109.5	H65B—C65—H65C	109.5
C08—C16—H16A	109.5	C58—C66—H66A	109.5
C08—C16—H16B	109.5	C58—C66—H66B	109.5
H16A—C16—H16B	109.5	H66A—C66—H66B	109.5
C08—C16—H16C	109.5	C58—C66—H66C	109.5
H16A—C16—H16C	109.5	H66A—C66—H66C	109.5
H16B—C16—H16C	109.5	H66B—C66—H66C	109.5
O23—C17—O14	109.6 (2)	O73—C67—O64	109.6 (2)
O23—C17—C18	107.5 (2)	O73—C67—C68	108.2 (2)
O14—C17—C18	110.4 (2)	O64—C67—C68	109.7 (2)
O23—C17—H17A	109.8	O73—C67—H67A	109.8
O14—C17—H17A	109.8	O64—C67—H67A	109.8
C18—C17—H17A	109.8	C68—C67—H67A	109.8
O24—C18—C19	107.6 (2)	O74—C68—C69	108.4 (2)
O24—C18—C17	110.7 (2)	O74—C68—C67	111.1 (2)
C19—C18—C17	109.9 (2)	C69—C68—C67	109.9 (2)
O24—C18—H18A	109.5	O74—C68—H68A	109.1
C19—C18—H18A	109.5	C69—C68—H68A	109.1
C17—C18—H18A	109.5	C67—C68—H68A	109.1
O28—C19—C18	109.2 (2)	O78—C69—C68	110.5 (2)
O28—C19—C20	108.5 (2)	O78—C69—C70	108.4 (2)
C18—C19—C20	109.3 (2)	C68—C69—C70	109.1 (2)
O28—C19—H19A	109.9	O78—C69—H69A	109.6
C18—C19—H19A	109.9	C68—C69—H69A	109.6
C20—C19—H19A	109.9	C70—C69—H69A	109.6
O32—C20—C19	107.5 (2)	O82—C70—C69	108.8 (2)
O32—C20—C21	106.5 (2)	O82—C70—C71	107.7 (2)
C19—C20—C21	110.7 (2)	C69—C70—C71	110.4 (2)
O32—C20—H20A	110.7	O82—C70—H70A	110.0
C19—C20—H20A	110.7	C69—C70—H70A	110.0
C21—C20—H20A	110.7	C71—C70—H70A	110.0
O23—C21—C22	106.2 (2)	O73—C71—C72	106.5 (2)
O23—C21—C20	109.8 (2)	O73—C71—C70	110.1 (2)
C22—C21—C20	111.7 (2)	C72—C71—C70	111.3 (2)
O23—C21—H21A	109.7	O73—C71—H71A	109.6
C22—C21—H21A	109.7	C72—C71—H71A	109.6
C20—C21—H21A	109.7	C70—C71—H71A	109.6
O36—C22—C21	111.0 (2)	O86—C72—C71	111.0 (2)
O36—C22—H22A	109.4	O86—C72—H72A	109.4
C21—C22—H22A	109.4	C71—C72—H72A	109.4
O36—C22—H22B	109.4	O86—C72—H72B	109.4
C21—C22—H22B	109.4	C71—C72—H72B	109.4
H22A—C22—H22B	108.0	H72A—C72—H72B	108.0
C17—O23—C21	114.2 (2)	C67—O73—C71	114.6 (2)
C25—O24—C18	115.6 (2)	C75—O74—C68	116.2 (2)
O26—C25—O24	123.3 (3)	O76—C75—O74	121.9 (3)
O26—C25—C27	125.7 (3)	O76—C75—C77	126.6 (3)
O24—C25—C27	111.1 (3)	O74—C75—C77	111.5 (3)
C25—C27—H27A	109.5	C75—C77—H77A	109.5
C25—C27—H27B	109.5	C75—C77—H77B	109.5
H27A—C27—H27B	109.5	H77A—C77—H77B	109.5
C25—C27—H27C	109.5	C75—C77—H77C	109.5
H27A—C27—H27C	109.5	H77A—C77—H77C	109.5
H27B—C27—H27C	109.5	H77B—C77—H77C	109.5
C29—O28—C19	116.9 (2)	C79—O78—C69	117.1 (2)
O30—C29—O28	124.1 (3)	O80—C79—O78	123.5 (3)
O30—C29—C31	124.9 (3)	O80—C79—C81	125.5 (3)
O28—C29—C31	111.1 (2)	O78—C79—C81	111.0 (2)
C29—C31—H31A	109.5	C79—C81—H81A	109.5
C29—C31—H31B	109.5	C79—C81—H81B	109.5
H31A—C31—H31B	109.5	H81A—C81—H81B	109.5
C29—C31—H31C	109.5	C79—C81—H81C	109.5
H31A—C31—H31C	109.5	H81A—C81—H81C	109.5
H31B—C31—H31C	109.5	H81B—C81—H81C	109.5
C33—O32—C20	118.1 (2)	C83—O82—C70	117.3 (2)
O35—C33—O32	123.9 (3)	O85—C83—O82	123.9 (3)
O35—C33—C34	125.9 (3)	O85—C83—C84	125.5 (3)
O32—C33—C34	110.2 (3)	O82—C83—C84	110.6 (2)
C33—C34—H34A	109.5	C83—C84—H84A	109.5
C33—C34—H34B	109.5	C83—C84—H84B	109.5
H34A—C34—H34B	109.5	H84A—C84—H84B	109.5
C33—C34—H34C	109.5	C83—C84—H84C	109.5
H34A—C34—H34C	109.5	H84A—C84—H84C	109.5
H34B—C34—H34C	109.5	H84B—C84—H84C	109.5
C37—O36—C22	117.4 (2)	C87—O86—C72	117.7 (3)
O39—C37—O36	122.8 (3)	O89—C87—O86	122.5 (3)
O39—C37—C38	126.3 (3)	O89—C87—C88	126.2 (4)
O36—C37—C38	110.9 (3)	O86—C87—C88	111.2 (4)
C37—C38—H38A	109.5	C87—C88—H88A	109.5
C37—C38—H38B	109.5	C87—C88—H88B	109.5
H38A—C38—H38B	109.5	H88A—C88—H88B	109.5
C37—C38—H38C	109.5	C87—C88—H88C	109.5
H38A—C38—H38C	109.5	H88A—C88—H88C	109.5
H38B—C38—H38C	109.5	H88B—C88—H88C	109.5
			
C02B—O01—C02A—C12A	48 (11)	C59—O51—C52A—C61A	−85.3 (10)
C09—O01—C02A—C12A	−162.9 (3)	C52B—O51—C52A—C61A	−114 (12)
C02B—O01—C02A—C03A	169 (12)	C59—O51—C52A—C62A	155.7 (7)
C09—O01—C02A—C03A	−42.6 (5)	C52B—O51—C52A—C62A	127 (12)
C02B—O01—C02A—C11A	−70 (11)	C59—O51—C52A—C53A	35.6 (15)
C09—O01—C02A—C11A	78.9 (4)	C52B—O51—C52A—C53A	7(11)
O01—C02A—C03A—C04A	59.6 (5)	O51—C52A—C53A—C54A	−53.6 (13)
C12A—C02A—C03A—C04A	175.9 (4)	C61A—C52A—C53A—C54A	61.1 (17)
C11A—C02A—C03A—C04A	−58.6 (5)	C62A—C52A—C53A—C54A	−175.7 (9)
C02A—C03A—C04A—C10	−45.5 (5)	C52A—C53A—C54A—C60	42.3 (12)
C09—O01—C02B—C11B	−95.5 (14)	C52A—O51—C52B—C61B	−120 (12)
C02A—O01—C02B—C11B	−62 (11)	C59—O51—C52B—C61B	86.5 (9)
C09—O01—C02B—C03B	18 (3)	C52A—O51—C52B—C53B	118 (12)
C02A—O01—C02B—C03B	52 (10)	C59—O51—C52B—C53B	−34.8 (16)
C09—O01—C02B—C12B	150.1 (16)	C52A—O51—C52B—C62B	−3(11)
C02A—O01—C02B—C12B	−176 (13)	C59—O51—C52B—C62B	−156.2 (7)
O01—C02B—C03B—C04B	−46 (3)	C61B—C52B—C53B—C54B	−56.8 (17)
C11B—C02B—C03B—C04B	56 (3)	O51—C52B—C53B—C54B	56.1 (16)
C12B—C02B—C03B—C04B	178 (2)	C62B—C52B—C53B—C54B	173.9 (11)
C02B—C03B—C04B—C10	40 (3)	C52B—C53B—C54B—C60	−41.4 (12)
C10—C05—C06—O14	−179.9 (2)	C60—C55—C56—C57	−6.7 (4)
C13—C05—C06—O14	−2.2 (4)	C63—C55—C56—C57	171.5 (2)
C10—C05—C06—C07	−3.9 (4)	C60—C55—C56—O64	178.2 (2)
C13—C05—C06—C07	173.9 (2)	C63—C55—C56—O64	−3.7 (4)
C05—C06—C07—C08	4.5 (4)	C55—C56—C57—C58	6.4 (4)
O14—C06—C07—C08	−179.3 (2)	O64—C56—C57—C58	−178.4 (2)
C05—C06—C07—C15	−172.1 (2)	C55—C56—C57—C65	−170.3 (2)
O14—C06—C07—C15	4.1 (3)	O64—C56—C57—C65	4.9 (4)
C06—C07—C08—C09	−1.2 (4)	C56—C57—C58—C59	−1.4 (4)
C15—C07—C08—C09	175.5 (2)	C65—C57—C58—C59	175.4 (3)
C06—C07—C08—C16	179.7 (2)	C56—C57—C58—C66	−178.5 (2)
C15—C07—C08—C16	−3.5 (4)	C65—C57—C58—C66	−1.7 (4)
C02B—O01—C09—C10	16.6 (15)	C52A—O51—C59—C58	175.5 (9)
C02A—O01—C09—C10	12.4 (4)	C52B—O51—C59—C58	179.4 (9)
C02B—O01—C09—C08	−166.3 (15)	C52A—O51—C59—C60	−4.2 (10)
C02A—O01—C09—C08	−170.5 (3)	C52B—O51—C59—C60	−0.3 (10)
C07—C08—C09—O01	−179.7 (2)	C57—C58—C59—O51	177.2 (3)
C16—C08—C09—O01	−0.7 (4)	C66—C58—C59—O51	−5.7 (4)
C07—C08—C09—C10	−2.7 (4)	C57—C58—C59—C60	−3.1 (4)
C16—C08—C09—C10	176.4 (3)	C66—C58—C59—C60	174.0 (3)
O01—C09—C10—C05	−179.9 (2)	O51—C59—C60—C55	−177.5 (3)
C08—C09—C10—C05	3.3 (4)	C58—C59—C60—C55	2.8 (5)
O01—C09—C10—C04A	2.4 (4)	O51—C59—C60—C54A	−7.9 (6)
C08—C09—C10—C04A	−174.5 (3)	C58—C59—C60—C54A	172.5 (5)
O01—C09—C10—C04B	−20.6 (15)	O51—C59—C60—C54B	13.7 (6)
C08—C09—C10—C04B	162.6 (15)	C58—C59—C60—C54B	−165.9 (4)
C06—C05—C10—C09	0.0 (4)	C56—C55—C60—C59	1.9 (4)
C13—C05—C10—C09	−177.8 (3)	C63—C55—C60—C59	−176.2 (3)
C06—C05—C10—C04A	177.8 (3)	C56—C55—C60—C54A	−167.1 (4)
C13—C05—C10—C04A	0.0 (4)	C63—C55—C60—C54A	14.7 (6)
C06—C05—C10—C04B	−158.1 (15)	C56—C55—C60—C54B	171.2 (4)
C13—C05—C10—C04B	24.2 (15)	C63—C55—C60—C54B	−6.9 (5)
C03A—C04A—C10—C09	14.9 (5)	C53A—C54A—C60—C59	−13.4 (8)
C03A—C04A—C10—C05	−162.8 (3)	C53A—C54A—C60—C55	155.9 (5)
C03A—C04A—C10—C04B	97 (3)	C53A—C54A—C60—C54B	−124 (2)
C03B—C04B—C10—C09	−9(3)	C53B—C54B—C60—C59	8.7 (8)
C03B—C04B—C10—C05	150.0 (15)	C53B—C54B—C60—C55	−160.2 (4)
C03B—C04B—C10—C04A	−118 (4)	C53B—C54B—C60—C54A	89 (2)
C05—C06—O14—C17	−68.2 (3)	C55—C56—O64—C67	−69.3 (3)
C07—C06—O14—C17	115.6 (3)	C57—C56—O64—C67	115.3 (3)
C06—O14—C17—O23	137.0 (2)	C56—O64—C67—O73	136.3 (2)
C06—O14—C17—C18	−104.8 (3)	C56—O64—C67—C68	−105.0 (2)
O23—C17—C18—O24	179.22 (19)	O73—C67—C68—O74	−179.99 (19)
O14—C17—C18—O24	59.8 (3)	O64—C67—C68—O74	60.5 (3)
O23—C17—C18—C19	60.5 (3)	O73—C67—C68—C69	60.0 (3)
O14—C17—C18—C19	−59.0 (3)	O64—C67—C68—C69	−59.4 (3)
O24—C18—C19—O28	64.1 (3)	O74—C68—C69—O78	61.9 (3)
C17—C18—C19—O28	−175.3 (2)	C67—C68—C69—O78	−176.5 (2)
O24—C18—C19—C20	−177.3 (2)	O74—C68—C69—C70	−179.1 (2)
C17—C18—C19—C20	−56.7 (3)	C67—C68—C69—C70	−57.5 (3)
O28—C19—C20—O32	−71.9 (2)	O78—C69—C70—O82	−67.5 (3)
C18—C19—C20—O32	169.06 (19)	C68—C69—C70—O82	172.2 (2)
O28—C19—C20—C21	172.1 (2)	O78—C69—C70—C71	174.6 (2)
C18—C19—C20—C21	53.1 (3)	C68—C69—C70—C71	54.2 (3)
O32—C20—C21—O23	−169.9 (2)	O82—C70—C71—O73	−172.12 (19)
C19—C20—C21—O23	−53.3 (3)	C69—C70—C71—O73	−53.4 (3)
O32—C20—C21—C22	72.6 (3)	O82—C70—C71—C72	70.1 (3)
C19—C20—C21—C22	−170.8 (2)	C69—C70—C71—C72	−171.3 (2)
O23—C21—C22—O36	57.8 (3)	O73—C71—C72—O86	50.8 (3)
C20—C21—C22—O36	177.5 (2)	C70—C71—C72—O86	170.7 (2)
O14—C17—O23—C21	56.5 (3)	O64—C67—O73—C71	57.8 (3)
C18—C17—O23—C21	−63.5 (3)	C68—C67—O73—C71	−61.8 (3)
C22—C21—O23—C17	−178.6 (2)	C72—C71—O73—C67	180.0 (2)
C20—C21—O23—C17	60.5 (3)	C70—C71—O73—C67	59.1 (3)
C19—C18—O24—C25	−160.4 (2)	C69—C68—O74—C75	−161.9 (2)
C17—C18—O24—C25	79.4 (3)	C67—C68—O74—C75	77.3 (3)
C18—O24—C25—O26	4.1 (4)	C68—O74—C75—O76	1.4 (4)
C18—O24—C25—C27	−176.4 (2)	C68—O74—C75—C77	−179.6 (2)
C18—C19—O28—C29	−112.1 (3)	C68—C69—O78—C79	−105.6 (3)
C20—C19—O28—C29	128.8 (2)	C70—C69—O78—C79	134.9 (2)
C19—O28—C29—O30	0.0 (4)	C69—O78—C79—O80	−1.7 (4)
C19—O28—C29—C31	178.9 (2)	C69—O78—C79—C81	178.5 (2)
C19—C20—O32—C33	103.3 (3)	C69—C70—O82—C83	99.7 (3)
C21—C20—O32—C33	−138.0 (2)	C71—C70—O82—C83	−140.7 (2)
C20—O32—C33—O35	6.1 (4)	C70—O82—C83—O85	7.9 (4)
C20—O32—C33—C34	−174.2 (3)	C70—O82—C83—C84	−172.3 (2)
C21—C22—O36—C37	79.0 (3)	C71—C72—O86—C87	78.2 (3)
C22—O36—C37—O39	2.3 (4)	C72—O86—C87—O89	4.3 (4)
C22—O36—C37—C38	−176.6 (2)	C72—O86—C87—C88	−174.4 (3)
